# Reviewed article: Soares LNS, Cabral AEA, Luz SBA, Lourenço MAG, Pazinatto RB, Santos BSM, Melo LA. Sociodemographic, general health and oral health factors associated with difficulty in eating among elderly people in Brazil: a cross-sectional study, 2019. Epidemiol Serv Saude. 2025:34;e20240678

**DOI:** 10.1590/S2237-96222025v34e20240678.a

**Published:** 2025-08-04

**Authors:** Ana Lúcia Schaefer Ferreira de Mello

**Affiliations:** Universidade Federal de Santa Catarina, Programa de Pós-graduação em Odontologia

**Table:** 

Rating	Excellent	Good	Average	Below average	Poor
Interest			✓		
Quality			✓		
Originality			✓		
Overall			✓		

**Table:** 

Questionnaire	Yes	No	Not applicable
Does the manuscript contain new and significant information to justify publication?	✓		
Does the Abstract (Summary) clearly and accurately describe the content of the article?		✓	
Is the problem significant and concisely stated?		✓	
Are the methods described comprehensively?	✓		
Are the interpretations and conclusions justified by the results?		✓	
Is adequate reference made to other work in the field?		✓	

## Manuscript Structure

Length of article is: Too short

Number of tables is: Adequate

Number of figures is: Adequate

Please state any conflict(s) of interest that you have in relation to the review of this paper (state “none” if this is not applicable): None

## Comments

Dear authors,

The manuscript deals with a relevant issue by investigating the factors associated with eating difficulties in older Brazilian adults, considering PNS 2019 data. Is essential to understand this phenomenon in the context of Brazilian health services. However, the results are too descriptive, and a deeper analysis is needed to improve the text/argument. Also, it is recommended that the practical implications for health services, health professionals, families, and older people be further presented. It also needs grammar review and literature update. The detailed revision is attached. 

